# Case Report: Bilateral symmetrical primary kaposiform hemangioendothelioma of the femur

**DOI:** 10.3389/fped.2022.995340

**Published:** 2022-11-04

**Authors:** Tong Qiu, Yuru Lan, Jiangyuan Zhou, Kaiying Yang, Xue Gong, Zixin Zhang, Siyuan Chen, Yi Ji

**Affiliations:** ^1^ Division of Oncology, Department of Pediatric Surgery, West China Hospital of Sichuan University, Chengdu, China; ^2^ Med-X Center for Informatics, Sichuan University, Chengdu, China; ^3^ Pediatric Intensive Care Unit, Department of Critical Care Medicine, West China Hospital of Sichuan University, Chengdu, China

**Keywords:** kaposiform hemangioendothelioma, bone, case report, Kasabach–Merritt phenomenon, pediatric

## Abstract

Kaposiform hemangioendothelioma (KHE) is a rare borderline vascular tumor that usually presents as a mass of skin or deep soft tissue. We report a unique case of an 8-year-old KHE patient with bilateral symmetrical sites involving both femurs. The laboratory, radiographic, and pathological findings of the patient were minutely described. During the 6-month follow-up, the symptoms of pain and dysfunction of this patient were relieved. This study aimed to arouse clinicians’ concern about the symmetrical sites of KHE patients.

## Introduction

Kaposiform hemangioendothelioma (KHE) is a rare borderline tumor with locally invasive features, which are mostly seen in children and adolescents, with an incidence of 0.71/100,000 (1). Clinical manifestations are more involved in the skin of the purplish red hard mass, and the surrounding tissue clearance is not clear. Only 10% of KHE patients showed deep lesions without skin involvement, while primary bone lesions were rarer, and most of the lesions were single lesions (2, 3). We report a unique case of KHE symmetrically involving both femurs, accompanied by pain and claudication, who was misdiagnosed as synovitis at an early stage. We aimed to alert clinicians to the early identification of KHE patients at specific sites, which is important for the management and prognosis of KHE patients.

## Case description

An 8-year-old female child was referred to our tertiary medical institution for pain and discomfort in both knees accompanied by claudication. Before admission to our hospital, she was treated for “joint synovitis” with anti-inflammatory treatment in a local hospital. The pain symptoms were relieved once, but the pain accompanied by claudication symptoms worsened 1 week after drug withdrawal. The patient showed local tenderness and percussion pain on both knees, but with no redness or palpable mass. Additionally, she showed no abnormal vital signs, such as fever and weight loss.

Laboratory tests, including white blood cell count of 12.56 × 10^9^/L (reference value was 4.3 × 10^9^/L–11.3 × 10^9^/L), D-dimer of 0.90 mg/L FEU (reference value was lower than 0.55 mg/L FEU), and C-reactive protein of 8.24 mg/L (reference value was lower than 5.00 mg/L), were mildly abnormal. Other tests, such as blood count, coagulation function, biochemical electrolyte, various metabolic indicators (including thyroid hormone- and bone metabolism-related indicators), erythrocyte sedimentation rate, antigen indicators of parasites, and various immune antibody indicators, were negative.

**Figure 1:**
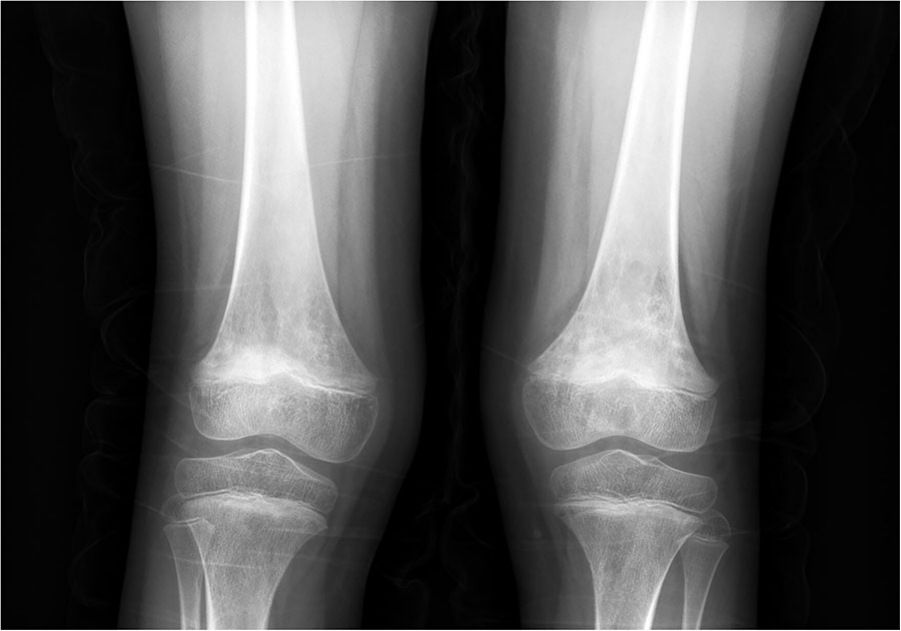
X-ray of both knee joints indicated uneven bone density in bilateral femoral diaphysis, epiphysis, and proximal tibia, with multiple low-density sites and calcification.

**Figure 2:**
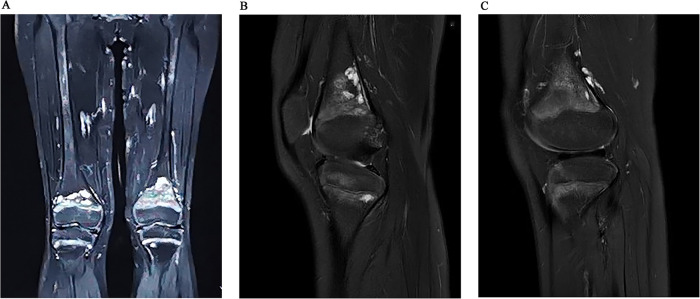
Coronal (**A**) and sagittal (**B**) MRI images indicated that lump-shaped shadow with long T1 and T2 signal was observed in the bottom of the femurs and in the upper end of tibias on both sides, no abnormality was observed in the shape and signal of the medial and lateral meniscus. After 6 months follow-up, we could see that the lesion was smaller than before (**C**).

**Figure 3:**
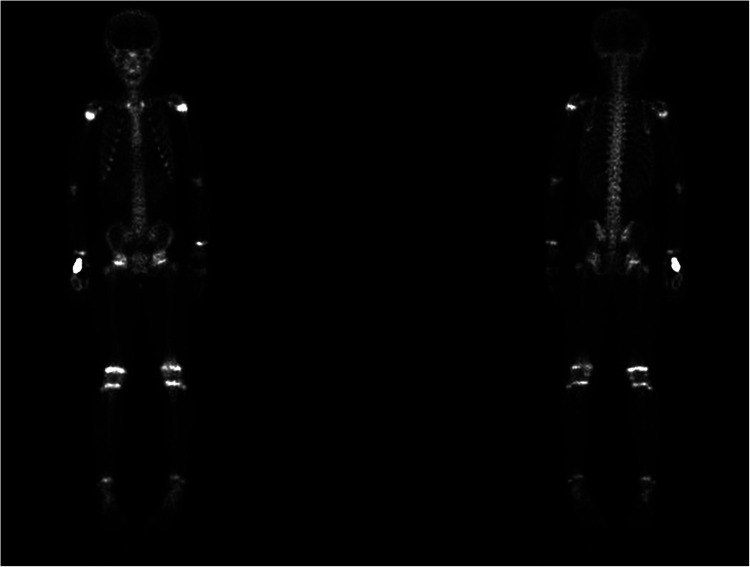
Whole-body bone imaging showed increased radiation in bone metabolism of bilateral lower femurs after intravenous 10 mCi of 99 mTc-MDP.

Imaging examinations included bilateral knee x-rays, computed tomography (CT) scans, magnetic resonance imaging (MRI), and whole-body bone imaging (Figures 1–3). Plain radiography and CT of both knee joints indicated uneven bone density in the bilateral femoral diaphysis, epiphysis, and proximal tibia, with multiple low-density sites and calcification. However, the joint space showed no obvious narrowing. MRI indicated that lump-shaped shadows with long T1 and T2 signals were observed in the bottom of the femurs and in the upper end of the tibias on both sides, and no abnormalities were observed in the shape and signal of the medial and lateral meniscus. No abnormalities were observed in the anterior and posterior cruciate ligaments or the tibial and fibular collateral ligaments. There was no fluid accumulation in the joint cavity and no swelling in the soft tissue around the joint. Whole-body bone imaging showed increased radiation in the bone metabolism of bilateral lower femurs after intravenous 10 mCi of 99 mTc-MDP.

**Figure 4:**
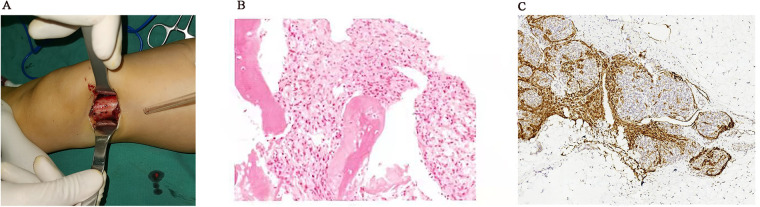
The left side of the distal femoral bone cortex was changing slightly rough in the operation (**A**), and the postoperative pathology results were showed (**B**: HE, ×200; **C**: positive D2-40, ×100).

To further clarify the patient's diagnosis, a biopsy operation opening the window of the left femur distal lesions and plaster external fixation were conducted after improving the preoperative examination. During the operation, we could see that the left side of the distal femoral bone cortex was changing slightly, and the surrounding tissue was not swollen. There was no obvious mass or edema around the periosteum, and yellow fat-like tissue could be seen in cancellous bone after opening the bone cortex window. Postoperative pathology results showed positivity for CD31, SMA, Ki-67, CD34, FA-8, ERG, and D2-40, and negativity for NSE (Figure 4). The above immunohistochemical and histological morphology results all supported the diagnosis of KHE.

After surgery, the patient was given oral sirolimus (0.8 mg/m^2^) monotherapy daily, and the blood concentration of sirolimus was maintained in the range of 3–8 ng/ml during the follow-up. Fortunately, the symptoms of claudication disappeared, and the hematological results completely returned to normal after a period of 6 months.

## Discussion

KHE can be divided into superficial, mixed, and deep types, and the most common one is the mixed type (4, 5). Nearly 70% of KHE cases can be accompanied by the Kasabach–Merritt phenomenon (KMP) of severe thrombocytopenia and coagulopathy (1). Although 62.8% of KHE patients were reported to have musculoskeletal diseases, most of the KHE lesions were deep soft tissue that invaded bones. Lesions primarily originating from bone and limited inside bone are very rare (6). Among a series of 31 KHE patients, Kuo et al. reported that there were six primary bone KHE cases involving the unilateral limb bones, spine, sacrum, scapula, and sternum (3). To the best of our knowledge, the KHE case involving bilateral femur symmetry reported in this study is the first to be reported in the literature world.

Among the 107 KHE patients reported by Croteau et al., only 3 cases were confined to bone without KMP (1). Additionally, in our case report, blood coagulation function was approximately normal, and KMP did not appear. This might be because the lesions are confined to the bone, and the soft tissue around the lower femurs on both sides was not involved. There was no periosteal reaction, and the lesions may be physically limited by the cortical bone, which could not be further invaded. The risk of KMP was smaller than that of skin and muscle lesions.

The most common lesions of KHE occur in limbs (7). However, in current studies on KHE, there were no cases with symmetric limbs involved and confined to the bone. Bilateral intraosseous lesions can be easily misattributed to osteomyelitis, fibrous dysplasia, and so on, which are caused by trauma, infection, or abnormal growth in clinical practice. The diagnosis of KHE depended on the comprehensive evaluation of clinical manifestations, hematology, imaging, and pathological results. The diagnosis of KHE can often be ignored and delayed for such rare symmetrical lesions, resulting in significant disability and mortality rates. Therefore, definite diagnosis through biopsy is particularly important for treatment direction, which can reduce the long-term complications of KHE.

Extensive resection of the lesion was not recommended for the KHE patient we reported to have bilateral femur lesions, which might lead to long-term complications and disability. Sirolimus is currently the first-line treatment for KHE (8–10). In our previous study, it was found that patients receiving sirolimus plus prednisolone therapy had fewer blood transfusions and a lower overall incidence of disease sequelae than those receiving sirolimus monotherapy. Sirolimus plus prednisolone is considered to be an effective treatment for KHE with KMP (2). We usually used sirolimus plus prednisolone to treat KHE patients with KMP, but for bone KHE patients without KMP, monotherapy with the mTOR inhibitor sirolimus acting on the PI3K/AKT/mTOR signaling pathway through inhibition of angiogenesis and lymphangiogenesis can effectively control and reduce mass and complications (11, 12).

## Conclusion

In conclusion, we should also be alert to the possibility of KHE of symmetry osteopathy in the clinic. Biopsy is the gold standard for clear diagnosis of this challenging disease. Early and timely diagnosis is crucial for the prognosis of complications such as pain and dysfunction and the quality of life of children.

## Data Availability

The original contributions presented in the study are included in the article/Supplementary Material, further inquiries can be directed to the corresponding author.
